# Severe Acute Respiratory Syndrome Coronavirus-2 Infection and Autoimmunity 1 Year Later: The Era of Vaccines

**DOI:** 10.3389/fimmu.2021.708848

**Published:** 2021-09-30

**Authors:** Andrea Picchianti Diamanti, Maria Manuela Rosado, Emanuele Nicastri, Giorgio Sesti, Claudio Pioli, Bruno Laganà

**Affiliations:** ^1^ Department of Clinical and Molecular Medicine, Sant’Andrea University Hospital, Sapienza University of Rome, Rome, Italy; ^2^ Research Consultant in Immunology, Rome, Italy; ^3^ Clinical Division of Infectious Diseases, Lazzaro Spallanzani, National Institute for Infectious Diseases-IRCCS, Rome, Italy; ^4^ Laboratory of Biomedical Technologies, Division of Health Protection Technologies, Italian National Agency for New Technologies, Energy and Sustainable Economic Development, Rome, Italy

**Keywords:** SARS-CoV-2, COVID-19, infections, vaccines, autoimmune rheumatic disease (ARD)

## Abstract

Impressive efforts have been made by researchers worldwide in the development of target vaccines against the novel severe acute respiratory syndrome coronavirus-2 (SARS-CoV-2) and in improving the management of immunomodulating agents. Currently, different vaccine formulations, such as viral vector, mRNA, and protein-based, almost all directed toward the spike protein that includes the domain for receptor binding, have been approved. Although data are not conclusive, patients affected by autoimmune rheumatic diseases (ARDs) seem to have a slightly higher disease prevalence, risk of hospitalization, and death from coronavirus disease-2019 (COVID-19) than the general population. Therefore, ARD patients, under immunosuppressive agents, have been included among the priority target groups for vaccine administration. However, specific cautions are needed to optimize vaccine safety and effectiveness in these patients, such as modification in some of the ongoing immunosuppressive therapies and the preferential use of mRNA other than vector-based vaccines. Immunomodulating agents can be a therapeutic opportunity for the management of COVID-19 patients; however, their clinical impact depends on how they are handled. To place in therapy immunomodulating agents in the correct window of opportunity throughout the identification of surrogate markers of disease progression and host immune response is mandatory to optimize patient’s outcome.

## Introduction

During the first wave of the coronavirus disease-2019 (COVID-19) pandemic, we discussed the complex relationship between autoimmunity and the novel severe acute respiratory syndrome coronavirus-2 (SARS-CoV-2) (1). At that moment, in the absence of specific antiviral treatments or available vaccines, we focused on the immunologic rationale and the initial scientific evidences for the effectiveness of immunomodulatory treatments, commonly adopted in autoimmune rheumatic diseases (ARDs), in the management of the aberrant hyper-inflammatory response induced by COVID-19. We concluded that anti-cytokine therapy, in particular the anti-IL-6 tocilizumab (TCZ) and the Janus kinase (JAK) inhibitor baricitinib, seemed to be the most promising drugs provided that their use was tailored to both timing of symptom onset and COVID-19 severity.

More than 1 year later, we are still facing a COVID-19 pandemic. However, combined adoption of universal containment measures and effective vaccine campaigns had a successful impact in terms of reduction of SARS-CoV-2 infections and COVID-19 hospitalizations. In addition, the use of immunosuppressive agents in the clinical management of COVID-19 patients has been clarified by consolidated data (2–4). First immunosuppressive agents, such as chloroquine and hydroxychloroquine (HCQ), have been discharged, whereas others have been better tailored to COVID-19 severity, timing of symptom onset, and association with other therapies. A considerable effort has been put forward to identify innovative vaccine platforms and to produce neutralizing monoclonal antibodies (mAbs) against SARS-CoV-2. In December 2020, the US Food and Drug Administration (FDA) and European Medicines Agency (EMA) approved the first mRNA vaccine (BNT162b2) against SARS-CoV-2, followed by other vaccines using mRNA platforms or inactivated viral vectors. Conversely, protein-based vaccines and conventional vaccines based on live attenuated or inactivated whole virus were left behind with still ongoing clinical trials or under approval by regulatory agencies.

The likely choice of use of different SARS-CoV-2 vaccines raised several questions and special concerns for its application in patients with ARD. First, ARD patients are part of vulnerable categories. Considering their intrinsic dysregulated immune system and the clinical impact of immunosuppressive treatments, a vaccine priority should be deserved. Second, safety and effectiveness of vaccination can be influenced by intrinsic vaccine formulations (mRNA *vs.* viral vectors), by disease-specific factors (ongoing immunosuppressive treatment) or by host factors (disease activity).

The aim of this review is to identify in the selected category of ARD patients the main controversial issues in terms of safety, efficacy, and effectiveness of SARS-CoV-2 vaccines and how to place vaccination in the context of the therapy with immunosuppressive agents, as well as to clinical parameters of disease severity and host immune response.

## Coronavirus Disease-19 Clinical Setting

To better understand the characteristics of the COVID-19 patients enrolled in randomized controlled trials (RCTs) evaluating the effectiveness and safety of immunosuppressive agents is fundamental to remind the generally accepted classification of disease severity (5).

Mild Infection (Approximately 80% of Cases): Individuals have signs and symptoms of COVID-19 (e.g., fever, cough, headache, muscle pain, nausea, diarrhea, and loss of taste and smell) with no evidence of viral pneumonia or hypoxia; they are usually outpatients.
Moderate Infection: Individuals show evidence of pneumonia during clinical assessment or imaging and have saturation of oxygen (SpO_2_) ≥92%–94% on room air at sea level; they are usually inpatients with no need of supplemental oxygen.
Severe Infection: Individuals have signs of severe pneumonia plus SpO_2_ <92%–94% on room air at sea level and/or a ratio of arterial partial pressure of oxygen to fraction of inspired oxygen (PaO_2_/FiO_2_) <300 mmHg, and/or respiratory rate >30 breaths/min; they are usually inpatients requiring high flow supplemental oxygen or mechanical ventilation.
Critical Infection (Approximately 5% of Cases): Individuals have acute respiratory distress syndrome (ARDS), with 100 mmHg < PaO_2_/FiO_2_a ≤ 300 mmHg, and acute life-threatening organ dysfunction and can experience cytokine storm syndrome (CSS) caused by a dysregulated host response; they are usually inpatients admitted in intensive care units requiring non-invasive (NIV) or invasive mechanical ventilation (IMV).

## Immunomodulating Anti-Rheumatic Agents Used in Severe Acute Respiratory Syndrome Coronavirus-2 Patients

### Hydroxychloroquine

HCQ is an oral drug frequently adopted in the treatment of systemic autoimmune diseases, with immunomodulatory effects and antiviral properties. The initial great enthusiasm for the potential benefits of this agent, sustained by some encouraging results from case series and retrospective studies (6), was subsequently denied by a deeper analysis of the data.

The FDA, EMA, and National Institutes of Health (NIH) have recommended against the use chloroquine or HCQ for COVID-19 patients (5).

### Colchicine

Colchicine is a well-known alkaloid agent currently approved in Italy for the treatment of gout and acute/recurrent pericarditis, but it is frequently used with effectiveness in the management of different autoinflammatory conditions (7, 8). It acts on several anti-inflammatory pathways, but the most recognized is the inhibition of microtubule polymerization, which can lead to impaired neutrophil migration, phagocytosis, and release of superoxide anion (9, 10). In addition, this agent limits neutrophil adhesion by both altering neutrophil L-selectin expression and E-selectin distribution in endothelial cells and inhibits Nod-Like Receptor Protein 3 (NLRP3) inflammasome known to be implicated in hyper-inflammatory syndrome (10, 11). These data provided the rationale for a possible role of this agent in the prevention of hyperinflammation and coagulation activation pathognomonic of COVID-19. Among RCTs, the GRECCO study showed that colchicine significantly improved time to clinical deterioration as compared with that in controls in hospitalized patients with moderate/severe COVID-19 not receiving ventilation support (12). In contrast, the RECOVERY trial closed the recruitment to the colchicine arm because there was no significant difference in the primary endpoint of 28-day mortality with respect to standard of care (SOC) in hospitalized COVID-19 patients (13). More recently, the largest RCT on colchicine in non-hospitalized COVID-19 patients (COLCORONA) did not meet the primary endpoint but demonstrated that in patients with PCR-confirmed COVID-19, colchicine led to a significant reduction in the composite rate of death or hospitalization (14).

In conclusion, there is no evidence for the efficacy of colchicine in hospitalized patients, and the drug is not included in the NIH recommendations. Its early use in outpatient settings to prevent hospitalization appears an attractive option to be confirmed.

## Interleukin Inhibitors

mAbs directed toward different inflammatory cytokines have been adopted in SARS-CoV-2 infection to reduce the aberrant immune response in COVID-19 patients with contrasting results. Most of available data are present for the humanized and human mAbs, recognizing the soluble and membrane-bound forms of IL-6 receptor, TCZ, and sarilumab, and also for anakinra, the non-glycosylated recombinant human IL-1Ra that blocks the binding of both IL-1α and IL-1β to its native receptor. IL-6 and IL-1 indeed play a central role in CSS (15–18) and are involved in several immunological activities such as the differentiation of naïve CD4-positive T cells into Th17 cells, increase acute-phase proteins and signaling, homing of immune cells to the site of primary infection, epithelial cell activation, and release of several other inflammatory cytokines (19, 20).

### Tocilizumab

TCZ was the first biologic agent to be used in severe COVID-19 patients also in view of the fact that it was the only approved biologic agent for the treatment of cytokine release syndrome (21, 22).

The first results from RCTs were unsatisfactory; however, the efforts in defining the right timing/segment of COVID-19 population combined with the concomitant use of a more effective SOC has made the difference in reaching the therapeutic targets.

Indeed, in all the first RCTs conducted, the COVACTA, the CORIMUNO-19 (23, 24), the Boston Area COVID-19 Consortium (BACC) Bay Tocilizumab (25), and the RCT by Salvarani et al. (2), TCZ did not show a significant improvement in clinical status or mortality reduction. Of note, these studies shared in common a very low use of glucocorticoid (GC) as SOC. Furthermore, in the COVACTA trial, more than two-thirds of participants were under mechanical ventilation.

The EMPACTA (Evaluating Minority Patients with Actemra, tocilizumab) was the first trial including a relevant percentage of patients receiving dexamethasone (DEX) and remdesivir (26). TCZ reached the composite primary outcome of progression to NIV/IMV, but without improving the survival rate.

More recently, the REMAP-CAP and RECOVERY, the two largest RCTs on TCZ, showed a significant efficacy of TCZ in mortality reduction in a well-defined COVID-19 population and in combination with DEX (27). Indeed, the REMAP-CAP enrolled severe/critical COVID-19 patients within 24 h after starting NIV or IMV, and most of the patients received concomitant GC. TCZ reduced both hospital mortality and time to hospital discharge. The RECOVERY trial enrolled severe COVID-19 hospitalized patients with hypoxemia, with or without mechanical ventilation, and C-reactive protein (CRP) level ≥75 mg/L. Patients on TCZ had a 4% mortality reduction through day 28, as well as the median time of hospitalization; the mortality benefit was observed in participants who were receiving concomitant GC. Considering all the above studies, NIH and WHO recommendations have recently stated that TCZ should be administered in combination with DEX in all severe/critical COVID-19 patients recently hospitalized (28).

### Sarilumab

Some small prospective studies explored COVID-19 patients receiving sarilumab with contrasting results on clinical outcomes, mortality, and time of hospitalization.

A phase 3 trial assessing 400 mg of sarilumab plus SOC *vs.* SOC alone, in critical COVID-19 patients (requiring IMV), was stopped because it did not meet its primary and secondary endpoints (29). In contrast with these results, the REMAP-CAP study reported median adjusted ORs for hospital survival at day 28 of 2.01 for i.v. sarilumab plus SOC *vs.* SOC alone (30). In July 2021, WHO recommends use of sarilumab with the same TCZ indications (5).

### Anakinra

A recent systematic review and meta-analysis reported data of seven studies on a total of 346 patients (31). These studies were different for design (four prospective and three retrospective), route and dose of anakinra administration, and population setting (mainly severe/critical COVID-19 patients); thus, results are hard to compare (32–38).

By now, only one RCT has been published on the use of anakinra in COVID-19 patients with disappointing results. It was a multicenter, open-label, Bayesian trial nested within the CORIMUNO-19 cohort, in patients with moderate-to-severe COVID-19, neither receiving mechanical ventilation nor admitted at ICU, and with a CRP >25 mg/L. Patients received either SOC plus i.v. anakinra at 400 mg/day for 3 days, 200 mg on day 4, and 100 mg on day 5, or SOC alone (39). The trial showed no significant difference between the two groups in terms of 4-day improvement, 14-day ventilation requirement, and 28-day mortality. The mean disease duration was 10 days before randomization, and the selected narrow segment target population (patients with a WHO score of 5 points and requiring oxygen, without mechanical ventilation) limits any conclusions.

Recently, the SAVE-MORE RCT was conducted in over 600 COVID-19 inpatients with elevated soluble urokinase plasminogen activator receptor (suPAR), a plasma biomarker that reflects immune activation. The comparative 11-point WHO Contracting & Procurement Services (CPS), at day 28, demonstrated significant improvement in patients receiving anakinra plus SOC *versus* SOC (40).

By now, the use of anakinra is not indicated by the NIH and WHO recommendations considering paucity of data; however, in July 2021, EMA started evaluating the use of anakinra in adult COVID-19 patients at increased risk of severe respiratory failure.

## JAK-STAT Inhibitors

JAK inhibitors are a class of orally administered targeted synthetic immunosuppressants that act by inhibiting the activity of one or more of the JAK members (JAK1, JAK2, JAK3, and TYK2), thereby interfering with the JAK-STAT signaling pathway and inhibiting the signaling of several inflammatory cytokines involved in autoimmunity diseases (41). Baricitinib reversibly inhibits JAK1/2-dependent cytokines (IL-6 and IFN-γ) and to a lesser extent JAK1/TYK2-dependent cytokines (IL-10 and IFN-α) (42).

Baricitinib has also shown to inhibit a regulator of endocytosis, the AP2-associated protein kinase 1 (AAK1), at therapeutic dosage for rheumatoid arthritis (RA); therefore, it can interrupt virus entry through receptor-mediated endocytosis (43). In addition, it has low interaction with the CYP drug-metabolizing enzymes, thus being safe in combination with antiviral drugs (44).

By now, the only RCT available is the Adaptive COVID-19 Treatment Trial 2 (ACTT-2) involving hospitalized patients with COVID-19 randomized to receive 4 mg/day of baricitinib plus remdesivir or remdesivir alone (45, 46).

Patients receiving baricitinib showed a significantly lower median time to recovery, with 30% higher odds of improvement in clinical status at day 15, and a similar incidence of serious adverse events (SAEs). The reduction in the time of hospitalization and the odds of improvement in clinical status were more clinically meaningful in the subgroup of patients with a baseline severity ordinal score of 6 (receiving high-flow oxygen or NIV) (47). The results of the CoV barrier in hospitalized COVID-19 patients who received once-daily baricitinib 4 mg plus SOC or SOC have been just released (unpublished preprint data). Treatment with baricitinib significantly reduced mortality in particular in subjects on high-flow oxygen/NIV, with a good safety profile (48).

The FDA currently approved the use of baricitinib in addition to remdesivir, in patients with moderate-to-severe COVID-19 requiring high-flow oxygen or NIV.

## Glucocorticoids

Most of the data come from the RECOVERY trial, the largest and first trial to be published. It demonstrated that the use of 6 mg of DEX daily up to 10 days plus SOC resulted in a significant decrease in 28-day mortality among patients who required mechanical ventilation or supplemental oxygen with respect to SOC (49). A systematic review and meta-analysis published in September 2020 selected six further RCTs evaluating the efficacy and safety of GCs in COVID-19 patients (50): the Efficacy of Dexamethasone Treatment for Patients With ARDS Caused by COVID-19 (DEXA-COVID 19) (51) and the COVID-19 Dexamethasone (CoDEX) trial, evaluated DEX in severe/critical COVID-19 patients under mechanical ventilation (52); the Randomized Embedded Multifactorial Adaptive Platform for Community-acquired Pneumonia (REMAP-CAP) (53), CAPECOVID (54), and COVIDSTEROID trial (55), evaluated hydrocortisone (HCT) in patients admitted to ICU or to an intermediate care unit who were receiving supplemental oxygen; finally, the Steroids-SARI trials was the only trial evaluating methylprednisolone (MTP) and enrolled patients admitted to ICU (56). The extent of concomitant SOC varied substantially among the trials. Overall death occurred in 32.7% of patients in GC group *vs.* 41.5% of SOC. The summary ORs for the association with all-cause mortality were 0.64 for DEX, 0.69 for HCT, and 0.91 for MTP. SAEs were reported by 18% of patients randomized to GC *vs.* 23% of patients in the control group (50).

In light of these data, DEX, alone or in combination with remdesivir, has become the SOC in severe and critical COVID-19 requiring supplemental oxygen with or without mechanical ventilation; alternatively, MTP or HCT can be adopted (52). It should be taken into account that GCs are not exempt from known severe side effects; in particular, high-dose GC can inhibit immune response, reduce pathogen clearance, and increase viral replication; thus, their use in mild/moderate patients not requiring oxygen supplementation and in the first stage of disease is contraindicated; moreover, low-dose (up to 6 mg of DEX or equivalent) and short-term GCs (up to 6 days) are recommended.

## Anti-Severe Acute Respiratory Syndrome Coronavirus-2 Monoclonal Antibodies

Before the COVID-19 pandemic, little attention had been paid to the use of mAbs, specific for pathogen epitopes, as therapy to treat infections. Despite, there are more than 100 mAbs that had been licensed as therapeutics for cancer, inflammation, and autoimmunity. The reasons behind the low interest to develop mAbs against infectious agents are mostly because of their high cost, which does not make them suitable for a broader use.

The high pressure generated from the SARS-CoV-2 pandemic induced first-line clinicians to explore the use of antibodies on the management of hospitalized patients. The initial approach was to transfuse plasma from previously infected convalescent individuals to hospitalized patients. Plasma samples were selected based on the titers of SARS-CoV-2-neutralizing antibodies, meaning that only individuals presenting high titers for the S1 subunit of SARS-CoV-2 spike protein, which contains the receptor-binding domain (RBD), were picked as donors. Such antibodies are able to prevent the entry of SARS-CoV-2 into cells by blocking the binding of the viral spike (S) protein to its cellular main receptor, angiotensin-converting enzyme 2 (ACE2) (57), or S-mediated membrane fusion. As plasma is obtained by apheresis, in addition to the SARS-CoV-2-neutralizing antibodies, each convalescent plasma (CP) preparation also contains the entire repertoire of antibody specificities, including natural antibodies, as well as proteins potentially working as immunomodulators (anti-inflammatory cytokines, clotting and anti-thrombotic factors, defensins, complement, and many others) (58). Actually, it is known that natural antibodies present in intravenous immunoglobulin (IVIg) preparations may sequester BAFF and reduce B-cell survival/proliferation or bind Fas and induce apoptosis, whereas exposure of dendritic cells to IVIg compromises dendritic cell (DC) maturation and downregulates costimulatory molecules such as CD86, CD80, and CD40 complement. IVIg also modulates the balance between CD4 and CD8 T cells and reduces the proliferation of Th17 cells as well as the secretion of inflammatory cytokines while promoting proliferation and survival of Treg cells (58). All the described immunomodulatory features of IVIgs are likely to be also present in the plasma of convalescent individuals; thus, CP therapy may benefit patients with COVID-19.

Because composition of CP is variable and patients receiving CP are under multiple concurrent therapies, it is not surprising that positive immunomodulatory effects of CP, on patients with COVID-19, diverge. In spite of that, plasma therapy was the first treatment authorized by the FDA for emergency use (40) based on trials demonstrating moderate benefit of plasma therapy, in particular when used promptly upon hospitalization, during the initial stages of disease, or in contexts in which patients are unable to produce their own antibodies against the coronavirus, such as in immunodeficient or in B-cell-depleted patients (40).

It is worthy to note that different studies reported low antibody responses in asymptomatic subjects and positive correlations between disease severity and intensity of the antibody response, with the highest Abs levels being observed in hospitalized patients. Moreover, B-cell receptor (BCR) sequence analysis revealed that SARS-CoV-2-neutralizing antibodies have few mutations as compared with their respective germline sequences (59, 60), suggesting poor germinal center reactions and thus a reduced capacity to induce long-lived plasma cells (61). These observations are extremely important for vaccine design and for the screening of neutralizing antibodies.

The generation of sub-neutralizing (or non-neutralizing) antibodies, in some infections, is responsible for antibody-dependent enhancement (ADE) of disease meaning that antibodies facilitate viral entry into host cells and enhance viral infections (62). ADE has been documented to occur through two distinct mechanisms in viral infections: by enhanced antibody-mediated virus uptake across Fc gamma receptor IIa (FcγRIIa)-expressing phagocytic cells leading to increased viral infection and replication or by excessive antibody Fc-mediated effector functions or immune complex formation, causing enhanced inflammation and immunopathology. All the viruses causing ADE have the ability to replicate in macrophages and/or alter their function. Although macrophages do not seem to be a major target for SARS-CoV-2, and the expression of ACE2 on different monocyte and macrophage populations is highly variable, previous data regarding SARS-CoV suggest that FcγRs can facilitate uptake of the virus into macrophages and B cells (63). While correlations between antibody titers and infection severity have been reported, the remaining question is whether the high antibody titers promote disease or whether severe infections elicit higher antibody titers (64).

In addition, sustaining a role for ADE in SARS-CoV *in vitro* studies showed that macrophages treated with serum from patients with SARS had exaggerated inflammatory cytokine profiles (65). It is worth noting that studies performed in 2014 by Wang and colleagues, during the first SARS infection, strongly suggested that SARS-CoV ADE is primarily mediated by anti-spike antibodies rather than antibodies against nucleocapsid proteins (66). This contributes to raise the concern for the use of CP without having done rigorous scientific studies as the ones done for other experimental treatments.

The ADE issue is important and has to be kept in mind during vaccine design and mAb production. The latter has been immediately activated by several academic laboratories (67) and pharmaceutical companies resulting in the synthesis of mAbs targeting SARS-CoV-2 spike protein RBD able to neutralize the virus at concentrations below 10 ng/ml (48). One of the first to be used was the LY-CoV555 (also known as LY3819253), developed by Eli Lilly after its discovery by researchers at AbCellera and at the Vaccine Research Center of the National Institute of Allergy and Infectious Diseases. This and other mAbs entered clinical testing within the first 6 months of the pandemic and showed to accelerate viral clearance and reduce hospitalization by 75% when administered early after infection (68). More recently, various groups are exploiting the possibility of combining mAbs (69), binding to different portions of the RBD, aiming at increasing the potency of protection but also preventing viral escape of immune responses due to viral mutations (70, 71).

In particular, two RCTs showed a significant reduction in hospitalizations and deaths among mild-to-moderate COVID-19 outpatient subjects receiving bamlanivimab plus etesevimab and casirivimab plus imdevimab compared with placebo. However, in both trials, the percentage of patients with hospitalization or death was also low in the placebo group (2.1% *vs.* 7.0% and 1% *vs.* 3.2%) (69, 72). Finally, the ACTIV-3 trial demonstrated that bamlanivimab plus remdesivir was not effective in hospitalized patients (73). Currently, the use of these mAbs is limited to outpatients with confirmed diagnosis of mild/moderate COVID-19 and specific risk factors for disease progression and hospitalization, within 10 days from the onset of symptoms. Nevertheless, UK RECOVERY investigators announced in June 2021 that high doses of casirivimab plus imdevimab reduced risk of death by 20% in patients hospitalized with COVID-19 who had not mounted their own immune response (74).

## Severe Acute Respiratory Syndrome Coronavirus-2 Vaccines

To date, several vaccines against SARS-CoV-2 have been approved by both the FDA and EMA and are currently being widely used in high-income countries. Several, of more than 70, vaccine candidates have reached the final stages for vaccine safety and protection efficacy in RCTs, but no direct comparative data between different vaccines or different platforms are available, and assays to titer viral neutralizing antibodies in the sera of vaccine recipients are not fully standardized. Theoretically a good vaccine, for any pathogenic agent, is a vaccine capable of reducing infection, disease, and transmission. The spike protein is required for SARS-CoV-2 receptor recognition, viral entry, and cell fusion and consequent infection. S protein, including its RBD, was shown to induce robust antibody response including generation of neutralizing antibodies, rendering it the target of almost all the approved and under-development vaccines against SARS-CoV-2. With the last decade technological achievements, vaccines can be produced on various platforms (75): viral vector, RNA, DNA, and protein-based in addition to the “traditional” inactivated and attenuated virus vaccines. These different types of vaccine induce immune responses through different mechanisms. Vaccines using adenoviral vector as antigen delivery are known to induce both cellular and humoral immunity already after a single immunization, with two immunizations raising a durable and long-lasting immune response (76, 77). Russia was the first country to approve a vaccine against COVID-19 named “Sputnik V,” formerly known as the “Gam-COVID-Vac.” This vaccine is based on a heterologous adenovirus strategy using two recombinant serotypically distinct adenovirus, namely, Adenovirus26 and Adenovirus5, both vectors including DNA coding for the spike protein. However, the world scientific community has raised doubts about the pivotal trial to prove its safety and efficacy, as its approval was obtained before public data disclosure, which only recently has been unleashed (78, 79). Published data showed that from 21 days after the first dose, the vaccine efficacy was 91.6% according to the number of confirmed COVID-19 cases.

Similar adenoviral vaccines are already used in large-scale vaccination programs all over the world. One of them is based on chimpanzee adenovirus called ChAdOx1 developed by the University of Oxford and AstraZeneca (80, 81), which showed a particularly high efficacy of 90.0% by inoculating two doses with a low first dose of vaccine (82). The other is an Adenovirus26 vector-based produced by Johnson & Johnson (83–85). Both vaccines are able to elicit potent and protective immune response, the latter even with a single dose (86). mRNA, FDA-approved vaccines first launched into the market were Pfizer/BioNTech and Moderna, which produced a viral modified mRNA encoding a more stable spike protein in a pre-fusion conformation form (87, 88). Because it is important for the immune system to respond to the virus before it enters into the cell, both Pfizer/BioNTech and Moderna vaccines deliver spike-specific mRNA into host cells through lipid nanoparticles (89).

The spike protein is synthesized translating the information encoded in the mRNA. The expression of SARS-CoV-2 spike protein elicits high titers of neutralizing antibodies and robust antigen-specific CD8^+^ and Th1-type CD4^+^ T-cell responses (88, 90). The efficacy rate in preventing COVID-19 infections for the Moderna (mRNA-1273) and Pfizer/BioNTech (BNT162b2) vaccines was 94.1% and 95.0%, respectively, with negligible side effects (91). The rapid emergence of SARS-CoV-2 variants, in particular the so-called UK and South African (SA) variants, carrying multiple mutations in the spike protein, raised the question whether mRNA vaccine-elicited antibodies were able to neutralize such variants. Neutralization geometric mean titers against N501Y from the United Kingdom and SA, 69/70-deletion/N501Y/D614G from the United Kingdom, and E484K/N501Y/D614G from SA variants of sera from subjects immunized with the BNT162b2 were 0.8- to 1.5-fold those against the parental virus (92). Noteworthy, these differences are much lower than those relative to the hemagglutination inhibition titers taken in account to consider a strain change in influenza vaccines (93). The virus-based vaccine candidates use weakened or inactivated virus processed by conventional technology by passing the virus through animal or human cells leading to mutations or by chemical substances (most commonly formaldehyde and heat) that make it less virulent.

Currently, two inactivated vaccines against SARS-CoV-2 were approved by at least one country. The first, Covaxin or BBV152, is a whole-virion inactivated SARS-CoV-2 vaccine (3 or 6 μg) formulated with a toll-like receptor 7/8 agonist molecule (IMDG) adsorbed to alum. It has been developed in India; and during the phase 1 trial, Covaxin was able to induce high neutralizing antibody responses that remained high in all participants at 3 months after the second vaccination. The phase 2 trial showed better reactogenicity and safety with enhanced humoral and cell-mediated immune responses compared with the phase 1 trial (94). The second vaccine has been developed in China by Sinopharm BBIBPCorV (95), which gives 79% protection (96).

Other whole virus-based vaccines with an inactivated virus, such as CoronaVac (97) and adsorbed vaccine COVID-19 by Sinovac (98), have just passed phase 3 clinical trials, and the sole live-attenuated COVI-VAC (developed in Russia, Codagenix Inc.) is in phase 1.

## Severe Acute Respiratory Syndrome Coronavirus-2 Vaccine in Rheumatic Autoimmune Diseases

### Rheumatic Autoimmune Diseases and Infections

Patients with ARD have an increased risk of infections, due to the immune-dysregulatory impact of the disease itself and the use of immunosuppressive agents.

Infections mainly involve bone and joints, skin, soft tissues, and respiratory tract; they are characterized by a more severe clinical outcome than in the general population and may induce disease flares, representing a frequent cause of death (99, 100).

In RA patients, the risk for serious infections is reported to be about double with respect to healthy subjects (101). Regarding common infections such as influenza, the reported incidence risk ratio (IRR) in RA patients *versus* general population is 1.2 (95% CI: 1.1–1.4), with a 2.75-fold increase in influenza-related complications (102). For *Streptococcus pneumoniae*, the reported IRR is 4.4 (95% CI: 3.8–5.2) in patients with RA and 4.3 (95% CI: 3.8–4.7) in patients with systemic lupus erythematosus (SLE), with an also increased risk of invasive pneumococcal disease (102). Beyond common risk factors (e.g., older age and comorbidities), the known specific risk factors for developing infections in ARD are mainly the concomitant immunosuppressive therapy, a high disease activity, and the presence of organ involvement (e.g., lungs, kidney, and leukopenia) (103–105). Regarding immunosuppressive treatments, it has been reported that the use of conventional synthetic disease-modifying antirheumatic drugs (csDMARDs) such as MTX and HCQ did not significantly affect the rate of influenza or its complications, whereas treatment with biological DMARDs (bDMARDs) are at a moderate higher risk than csDMARDs, with no significant differences among them. GC use over 7.5 mg/day in particular in combination with anti-TNF-α appears to be the most pro-infective treatment (104–108).

### Prevalence and Outcome of Severe Acute Respiratory Syndrome Coronavirus-2 Infection in Rheumatic Autoimmune Diseases

In our previous review, we hypothesized that the impairment of immune response caused by the ongoing therapy in ARD could be a double-edged sword in the context of COVID-19 pandemia.

On the one hand, indeed, immunosuppressive therapy could increase the risk of COVID-19, but on the other hand, the cytokine release could be restrained by the effect of the immunomodulating agents, thus potentially reducing COVID-19 symptoms and severity, and the risk of the aberrant inflammatory response observed in some critical infected patients.

More than 1 year later, interpretation of data on whether or not patients with ARD are at higher risk of COVID-19 prevalence and complications are still not straightforward due to the heterogenicity of autoimmune diseases analyzed, study design, ongoing therapeutic regimen, and disease activity.

A systematic review and meta-analysis published in September 2020 showed a COVID-19 prevalence of 0.009 (95% CI: 0.005–0.014) in patients with ARD (0.034 in the studies evaluating only SLE, Sjogren’s syndrome, and systemic sclerosis), by analyzing 33 observational studies with a total of 54,679 subjects (109). Regarding COVID-19 severity, the overall rates of hospitalization and death assessed by 34 studies in 1,449 patients with ARD were 0.54 (95% CI: 0.46–0.63) and 0.113 (95% CI: 0.098–0.13), respectively (0.33 and 0.069 in the studies evaluating only SLE, Sjogren’s syndrome, and systemic sclerosis). The six case–control studies demonstrated that the risk of COVID-19 in ARD was significantly higher than that in controls (OR: 1.59, 95% CI: 1.04 to 4.58, p = 0.008), whereas there were no significant differences in clinical outcomes, such as hospitalization and death.

Since then, several data have been published on a large population such as those from the COVID-19 Global Rheumatology Alliance (GRA) and from nationwide cohort studies and registries (110–113). Furthermore, another systematic review and meta-analysis evaluating 1,138 patients with various rheumatic diseases from 31 studies (16 of which were different from those included by Akiyama et al.) have recently reported that ARD represents a risk factor for poor outcomes in patients with COVID-19.

In particular, the rates of hospitalization and fatality among COVID-19-infected patients with ARD were 0.58 (95% CI: 0.48–0.67) and 0.07 (0.13 among hospitalized patients) (95% CI: 0.03–0.11), respectively (114).

In conclusion, although data are partly contrasting and hard to compare, ARD seem to have a similar or slightly higher prevalence, risk of hospitalization, and death from COVID-19 than the general population, as already stated by the last European League Against Rheumatism (EULAR) and American College of Rheumatology (ACR) recommendations (115, 116). As for the general population, older age, male gender, and comorbidities such as hypertension, diabetes, lung disease, and obesity have been described as independent risk factors associated with COVID-19 death and worse outcome. Whereas among disease-specific risk factors, the use of prednisolone-equivalent dosage >5/10 mg/day, some csDMARDs (e.g., azathioprine, cyclophosphamide (CyC), and mycophenolate), and rituximab (RTX) and having an active disease were the most frequently reported factors associated with poor prognosis. Although there is no clear evidence of worse outcome due to specific factors related to the disease itself other than GC use, also in SLE patients (117), the presence of kidney involvement should be taken into account considering that it is a known risk factor for severe disease and mortality in COVID-19 patients (118). On the other hand, MTX and bDMARD monotherapy (other than RTX) were reported to have a protective rather than negative effect on COVID-19 outcome. Data on targeted synthetic (ts)DMARDs are still contrasting and on small sample size (109, 110, 119).

### Efficacy and Safety of Common Vaccines in Rheumatic Autoimmune Diseases

The particular relevance of vaccinations in ARD patients is indisputable, considering that vaccines can lead to a lower prevalence of infections and related complications such as hospitalization and death. In the last years, the efforts of several groups gave important contributions in this field, generally overcoming prejudices that limited this good clinical practice in patients with ARD, such as the fear of an impaired efficacy (as a result of immunosuppression) and decreased safety (linked to a possible exacerbation of the underlying autoimmune disease).

Among the vaccine-preventable respiratory diseases, influenza and pneumococcal infections have been the more extensively studied in ARD patients. Last, EULAR recommendations stated that both these vaccines should be strongly considered for the majority of patients with ARD (120).

Indeed, as reported by a systematic review and meta-analysis, most studies evaluating immunogenicity (expressed as seroconversion rate; ≥4-fold increase in hemagglutinin titer) of inactivated influenza vaccine showed that autoimmune patients (mainly RA and SLE) reached protective antibody levels, although reduced with respect to healthy controls (121, 122). In particular, for the H1N1 strain, the pooled mean proportion of 11 studies on 883 RA patients was 50.5% *vs.* 67% of healthy controls (p = 0.05), whereas for H3N2 strain, the pooled mean proportion of eight studies on 333 patients was 54.7% *vs.* 50.2% (ns). The percentage of adverse events was similar to that of controls, and the disease activity was generally stable. The humoral immunogenicity of the 23-valent pneumococcal polysaccharide vaccine (PPSV23) and to a lesser extent for the 13-valent conjugate vaccine (PCV13) has been demonstrated for both RA and SLE patients with no significant safety concerns independently of the type of vaccine (120, 122).

### Efficacy and Safety of Severe Acute Respiratory Syndrome Coronavirus-2 Vaccine in Rheumatic Autoimmune Diseases

**Figure 1:**
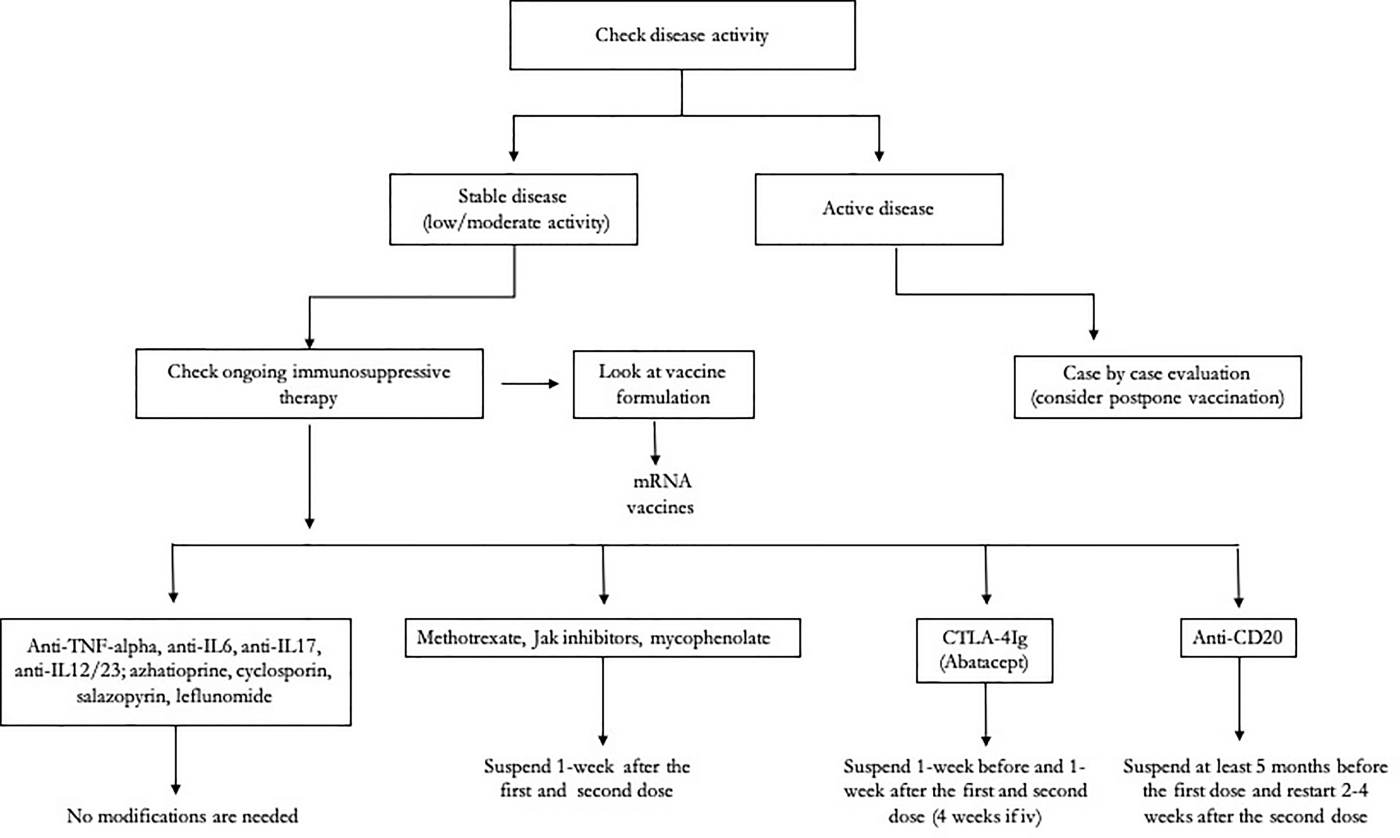
Clinical algorithm for the management of patients with autoimmune rheumatic diseases (ARDs) undergoing severe acute respiratory syndrome coronavirus-2 (SARS-CoV-2) vaccination.

In Italy, as well as in other countries, autoimmune patients under immunosuppressive therapy have been included among priority target groups for vaccine administration (123–125). While waiting for evidence-based data, at least three questions are currently a challenge for ARD (**Figure 1**):

- Is there a preferred vaccine for ARD patients?
- What is the effect of ongoing immunosuppressive therapy?
- Can disease activity influence or be influenced by SARS-CoV-2 vaccine?

### Influence of Vaccine Formulation

According to the previously cited ACR recommendations, there is no preference for one COVID-19 vaccine over another in ARD patients. Indeed, ARD patients were mainly excluded from clinical trials, so there were inadequate data to draw any conclusions on the efficacy and safety of COVID-19 vaccination in these patients. Nevertheless, different observational data are currently becoming available for mRNA vaccine in this patient category (**Table 1**), whereas we still have no data for viral vector vaccines; furthermore, some immunologic insights are tipping the scale in favor of mRNA-based vaccine. Indeed, is important to consider that after vector-based vaccines, you can respond not only to the spike but also to the adenovirus developing anti-vector antibodies and a lower spike-specific response, thus requiring frequent booster doses with a consequent increased risk of autoimmune flares. On the other hand, mRNA vaccines by reducing the toll-like receptor-7 stimulation could potentially attenuate the risk of disease flares in ARD patients (131).

**Table 1:** Core relevant data from studies evaluating efficacy and safety of SARS-CoV-2 vaccine in ARD patients.

	ARD Total number Subtype number	Type of vaccine	Age Mean, years (range)	Timing of assessment(weeks)	Seropositivity rate %(serum anti-S1 IgG titer, mean)								T-cell responses	Side effects/disease relapses
					Controls	Patients(therapy)								
						Tot	MTX	TNFi	IL6i	JAKi	CTLA4i	CD20i		
Furer et al. (126)	**Tot: 686** RA 263 PsA/SpA 233 SLE 101 Vasculitis 66 Other 23	BNT162b2	59 (19-88)	2–6 weeks from 2nd dose	100 (218.6)	86^*^ (132.9)	92	98	100	90	71	41	NA	Similar to HCs/stable in most pts
Geisen et al. (127)	**Tot: 26** RA 8 PsA/SpA 5 SLE 2 Other 11	BNT162b2	50.5 (24–89)	1 week from 2nd dose	100 (2,685)	100 (2,053^**^)	NR	NR	NR	NR	NR	NR	NA	Similar to HCs/none
Haberman et al. (128)	**Tot: 51** RA 22 PsA/PsO 24 Other 5	BNT162b2	56 (22–79)	1 week from 2nd dose	96 (104.3)	82 (80.9)	72^#^ (46.9)	NR	NR	NR	NR	NR	^Reduced in pts receiving MTX	NA
Simon et al. (129)	**Tot: 84** RA 24 SpA 27 SLE 16 Other 16	BNT162b2	53 (NA)	At least 10 days from 2nd dose	100 (9.36)	94^$^ (6.46)	NR	NR	NR	NR	NR	NR	NA	Similar to HCs/NA
Picchianti Diamanti et al. (130)	**Tot: 35 RA**	BNT 162b2	59 (55-65)	2–6 weeks from 2nd dose	167 (2351)	97 (785§)	100 (1526)	100 (1239)	100 (492§)	NR	92 (465§)	NA	Reduced in pts receiving TNFi, IL6i and CTLA4i	Similar to HCsNone

NA, not assessed; NR, not reported; ARD, autoimmune rheumatic disease; RA, rheumatoid arthritis; PsA, psoriatic arthritis; SpA, spondyloarthritis; SLE, systemic lupus erythematosus; MTX, methotrexate; TNFi, tumor necrosis factor inhibitors; JAKi, Janus kinase inhibitors; CD20i, CD20 inhibitors.

^*^p < 0.0001 (BAU/ml, cutoff = 15).

^**^p = 0.037 (BAU/ml, cutoff = above 2,000 but not specifically reported).

^#^p = 0.023 (median; cutoff = not specifically reported).

^$^p = 0.003 (optical density, cutoff = 5.7 nm).

^Evaluated by high-parameter spectral flow cytometry.

After small pivotal trials of the mRNA-based vaccine BNT162b2 in few ARD patients (127–129, 132), a larger study, in the Israel population, showed that the majority of the RA patients respond to the vaccine with a seropositivity rate of 86%, although with a lower humoral response than the control group. Overall, BNT162b2 showed a good safety and no significant disease flares (126).

Some immunosuppressive treatments, such as RTX, abatacept, mycophenolate mofetil (MMF), and methotrexate (MTX), showed negative impact on vaccine immunogenicity; however, data are partly contrasting and need to be evaluated by additional real-life studies (**Table 1**). In the attempt to provide useful insights, we have recently assessed the induction of specific humoral and T-cell response after mRNA BNT162b2 SARS-COV-2 vaccination in RA patients who underwent a strategy of temporary modification of immunosuppressive treatment according to ACR indications. The vaccine was safe and no disease relapses were observed. The vaccine induced an antibody-specific response in almost all patients, although the titer was significantly reduced in those under abatacept or IL-6-inhibitor compared to HCs. Spike-specific T-cell response was positive in 69% of RA patients vs. 100% of HCs with significantly lower levels in those under a biological therapy.Our data suggest that holding treatment with MTX and abatacept at the first and second vaccine dose can be a succesfull strategy in clinically stable patients (130).

In particular, the timing of vaccination related to the immunosuppressive therapy should be better defined, which could play a crucial role on the size of the response.

In particular, the timing of vaccination related to the immunosuppressive therapy should be better defined, which could play a crucial role on the size of the response.

### Influence of Immunosuppressive Agents

Whether to modify the ongoing immunosuppressive treatment in ARD patients who are to receive SARS-CoV-2 vaccinations can be challenging, because the balance of the risk of disease relapse with that of a decreased immunogenicity has to be weighed in. Until now, recommendations for SARS-CoV-2 vaccine in ARD have been formulated by the ACR (130, 133). The authors stated that ARD patients should be prioritized for vaccination and have no specific contraindications. However, expected response to COVID-19 vaccination for many patients on systemic immunomodulatory therapies is likely to be blunted in its magnitude and duration. The authors reported that most of the csDMARDs such as HCQ, ciclosporin, salazopyrin, azathioprine, leflunomide, oral CyC, and GC <20 mg/day do not require modifications in therapeutic regimen or vaccination timing. On the other hand, MTX, mycophenolate, JAK inhibitors, and i.v. CyC should be held 1 week after each vaccine dose, at least for patients with well-controlled disease. Among bDMARDs, anti-TNF-α, anti-IL-6R, anti-IL-1, anti-IL-17, anti-IL-12/23, and anti-BLYS do not require specific modifications on therapy frequency/dosage or vaccination timing, except for abatacept, which should be stopped 1 week prior to and 1 week after the first dose only (4 weeks for i.v. route) (**Figure 1**).

The anti-CD20 RTX deserves special clinical attention. Indeed, vaccine timing should be modified so that the first dose is initiated 4 weeks prior to next scheduled RTX cycle; moreover, after vaccination, RTX should be delayed 2–4 weeks after the second vaccine dose, if disease activity allows. These recommendations were previously released by the ACR Board of Directors in February 2021 and then published in June. Considering the almost absent available scientific evidence, they were based on data from common vaccinations. Indeed, regarding seasonal and pandemic influenza, and pneumococcal inactivated vaccines, the use of TNF-α-blocking agents, anti-IL-6, and csDMARDs (other than MTX), in the majority of studies did not induce a reduction of immunogenicity (120, 134–139). Fewer studies with small sample size evaluated the effect of abatacept and JAK inhibitors with contrasting data; however, most of them reported a mild reduction in humoral immunogenicity (140–143). By now, studies on SARS-CoV-2 mRNA vaccine in ARD patients have generally confirmed the neutral impact of cytokine inhibitors and most of the csDMARDs on vaccine immunogenicity, as well as a significant impairment caused by abatacept (126). Data on the impact of MTX in vaccine immunogenicity are more controversial. Indeed, no negative effect of MTX was found for influenza vaccination, in most studies including one meta-analysis in patients with RA (144–148). Only two studies by Park et al. in patients with RA showed that temporary discontinuation of MTX significantly improves immunogenicity to seasonal influenza vaccine (149, 150). In particular, suspension of MTX for 2 weeks after vaccination led to an 11%–16% (depending on the strain) higher seroprotection rate compared with that in RA patients who continued MTX. Two other studies reported a reduced pneumococcal humoral immunogenicity in patients receiving MTX (131, 151, 152). In line with these data, the 2019 EULAR recommendations for vaccination in ARD patients stated that it is not recommended to stop MTX before or after influenza and pneumococcal vaccines (120). These concerns have not been resolved by recently published data on COVID-19 mRNA vaccines in ARD patients who report contrasting results on the effect of MTX, ranging from a relevant to mild reduction in humoral response with respect to controls (**Table 1**). Regarding RTX, this agent has been associated with hampered antibody responses following influenza and pneumococcal vaccination in multiple studies, and the EULAR recommendations report that they should be scheduled at least 6 months after the administration and 4 weeks before the next course of anti-CD20 therapy (119). In these patients, B-cell depletion is due to different mechanisms such as antibody-dependent cell-mediated cytotoxicity, complement-dependent cytotoxicity, and apoptosis (153), whereas, it did not significantly affect T cell-mediated response in RA patients receiving influenza vaccine (154). Data from the 2003 SARS outbreak and the current SARS-CoV-2 pandemic showed the role of protective memory T-cell responses in recovered and asymptomatic individuals, with the emergence of memory T cells also in the absence of an antibody response (155–157). Indeed, asymptomatic people who have cleared SARS-CoV-2 can have a detectable specific T-cell response, without mounting an antibody response (157–160), observation confirmed by the recovery of people with B-cell immunodeficiency, or receiving B-cell-depleting therapy (161, 162). Furthermore, considering that most of the SARS-CoV-2 vaccines need a second dose after 3–4 weeks, this prolonged interruption of RTX could lead to an increased risk of disease relapse.

Current data confirm the severe reduction of humoral response to mRNA SARS-CoV-2 vaccine in patients under RTX, however accompanied by a substantial maintenance of the adaptive cellular immunity (163).

In view of the above, the immunologic response following SARS-CoV-2 vaccine in ARD patients receiving MTX and RTX deserves to be better investigated by additional real-life studies.

### Severe Acute Respiratory Syndrome Coronavirus-2 Vaccination and Disease Activity

Autoimmune diseases, infections, and vaccines are also joined with disease activity. A high disease activity is a poor prognostic factor for COVID-19 severity, and infections even by SARS-CoV-2 can induce disease flares in ARD. Vaccines could work in a similar manner, thus potentially inducing clinical relapses in ARD and having a reduced immunogenicity in active patients. Indeed, high disease activity can reflect an ongoing active inflammation against self, which could deplete the immune resources and partly deviate them from the signals delivered by vaccines (164); moreover, these patients are generally under a stronger immunosuppressive therapy that can further decrease immunogenicity. Based on these assumptions, few studies on common vaccination included patients with active disease. In one study, patients with juvenile SLE and high activity reduced seroconversion rates to influenza A H1N1 vaccination (165). Conversely, in a study of 340 RA patients, high disease activity levels did not reduce immunogenicity (166). Data are reassuring with regard to disease reactivation after common vaccines in ARD patients; almost all the studies, indeed, reported that vaccination did not influence activity of the underlying ARD such as RA and SLE (122, 167–174).

Concerns about the possible risk of disease relapse in these patient populations, related to the use of new vaccine technologies, have been reduced by recently published manuscripts on BNT162b2 vaccine in ARD patients who showed a good safety and no significant disease flares. Moreover, a cross-sectional study based on a web-based survey on 696 SLE participants reported that COVID-19 vaccination (both mRNA and adenoviral platforms) appears well tolerated with only a minimal risk of flare (3%) (175).

Anyway, considering the paucity of data on active patients, the EULAR recommendations report that vaccination in patients with ARD should be preferably administered during quiescent disease (120). In apparent contrast, the ACR recommendations stated that COVID-19 vaccination should occur as soon as possible for patients for whom it is being recommended, irrespective of disease activity and severity (130).

In conclusion, considering the severity of the SARS-CoV-2 infection, the current pandemic emergency, and the evidence from common vaccinations, the chance of protect also active patients seem to weight more in the balance than the possible risk of a reduced immunogenicity and safety concerns.

## Conclusions

Eighteen months after SARS-CoV-2 identification, we are still facing the COVID-19 pandemic despite all public health measures put in place. Nevertheless, the spread of the virus is being strongly reduced by vaccines based on various platforms currently adopted in most countries. Available data suggest that ARD patients have a similar or slightly higher prevalence of SARS-CoV-2 infections, risk of hospitalization, and death from COVID-19 than have the general population. They have been included among priority groups for vaccination, but special measures should be adopted to improve vaccine safety and effectiveness, throughout modification in ongoing immunosuppressive regimen and preferential use of mRNA-based vaccines as demonstrated by emerging data on mRNA vaccination efficacy in ARD patients.

Immunomodulating agents are an important therapeutic opportunity for the management of COVID-19 patients. To place therapy immunomodulating agents in the correct window of opportunity throughout the identification of surrogate markers of disease progression and host immune response is mandatory to optimize patient’s outcome.

## Author Contributions

AP and BL conceived the manuscript. AP, MR, and CP wrote the manuscript. EN and GS critically revised the manuscript. All authors contributed to the article and approved the submitted version.

## Funding

This work was supported by Progetto di Ateneo 256/2020 funded by "Sapienza" Università degli Studi di Roma, by Ricerca Corrente “Infezioni Emergenti e Riemergenti” and by Progetto COVID-2020-12371675 both funded by Italian Ministry of Health.

## Conflict of Interest

The authors declare that the research was conducted in the absence of any commercial or financial relationships that could be construed as a potential conflict of interest.

## Publisher’s Note

All claims expressed in this article are solely those of the authors and do not necessarily represent those of their affiliated organizations, or those of the publisher, the editors and the reviewers. Any product that may be evaluated in this article, or claim that may be made by its manufacturer, is not guaranteed or endorsed by the publisher.
